# Supplementary material for: ACL and HAT1 form a nuclear module to acetylate histone H4K5 and promote cell proliferation
Source: Nat Commun. 2023 Jun 5;14:3265. doi: 10.1038/s41467-023-39101-4 (PMC10241871; doi:10.1038/s41467-023-39101-4)
Supplement: Supplementary file 1 — Supplementary Information [file 41467_2023_39101_MOESM1_ESM.pdf]

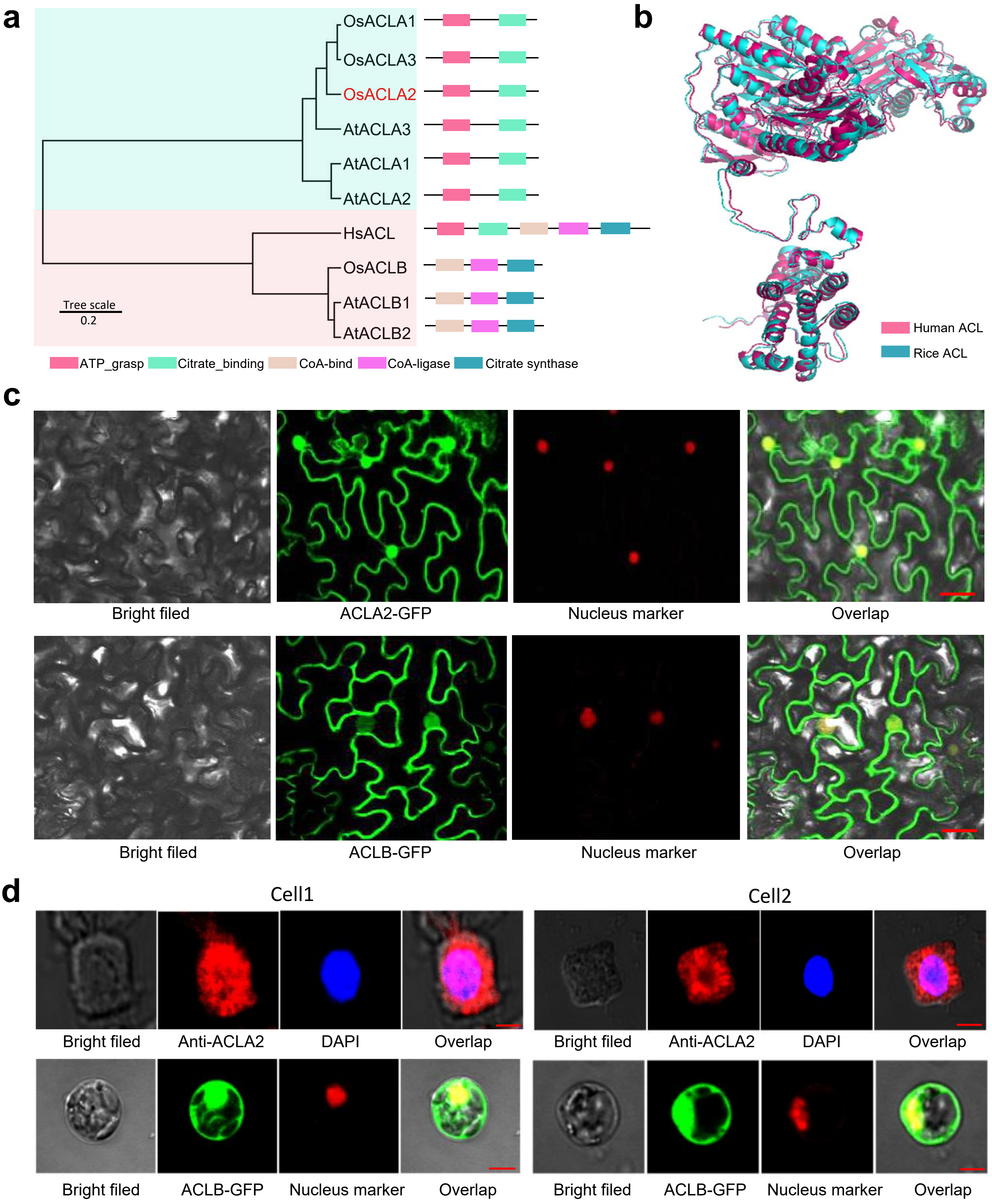

Supplementary Fig. 1. Phylogeny, 3D structure modeling, and subcellular localization of rice ACL proteins. a Phylogenetic tree of ACL (ATP citrate lyase) family members from *Arabidopsis* (At), *Oryza sativa* (Os), and *Homo sapiens* (Hs) generated by MEGA5 using the neighbor joining method. ACLA and ACLB subfamilies were colored as cyan and light red, respectively. The scale bar indicates the average number of amino acid substitutions per site. **b** Comparison of 3D modeling of human (pink) and rice (cyan) ACL. The rice ACL is composed of ACLA2 and ACLB. **c** Subcellular localization of ACLA2 (upper) and ACLB (lower) proteins. Cytoplasmic and nuclear localization of ACLA2 and ACLB were observed in tobacco leaf cells. ACLA2 or ACLB coding sequence was fused to GFP at its C-terminus and placed under the control of the cauliflower mosaic virus 35S promoter. RFP signals indicate the nuclei. Scale bars = 30 μm. The experiments were repeated three times with similar results, and representative data are presented. **d** Subcellular localization of ACLA2 (upper) and ACLB (lower) proteins. Upper: Immunofluorescent staining of wild type rice root tip cell with anti-ACLA2 antibody. Nucleus (blue) was stained with DAPI. Lower: Cytoplasmic and nuclear localization of ACLB in rice protoplasts. ACLB coding sequence was fused to GFP at its C-terminus and placed under the control of the its promoter (2kb upstream of the start codon). RFP signal indicates the nucleus. Scale bars = 5 μm. The experiments were repeated three times with similar results, and representative data are presented.

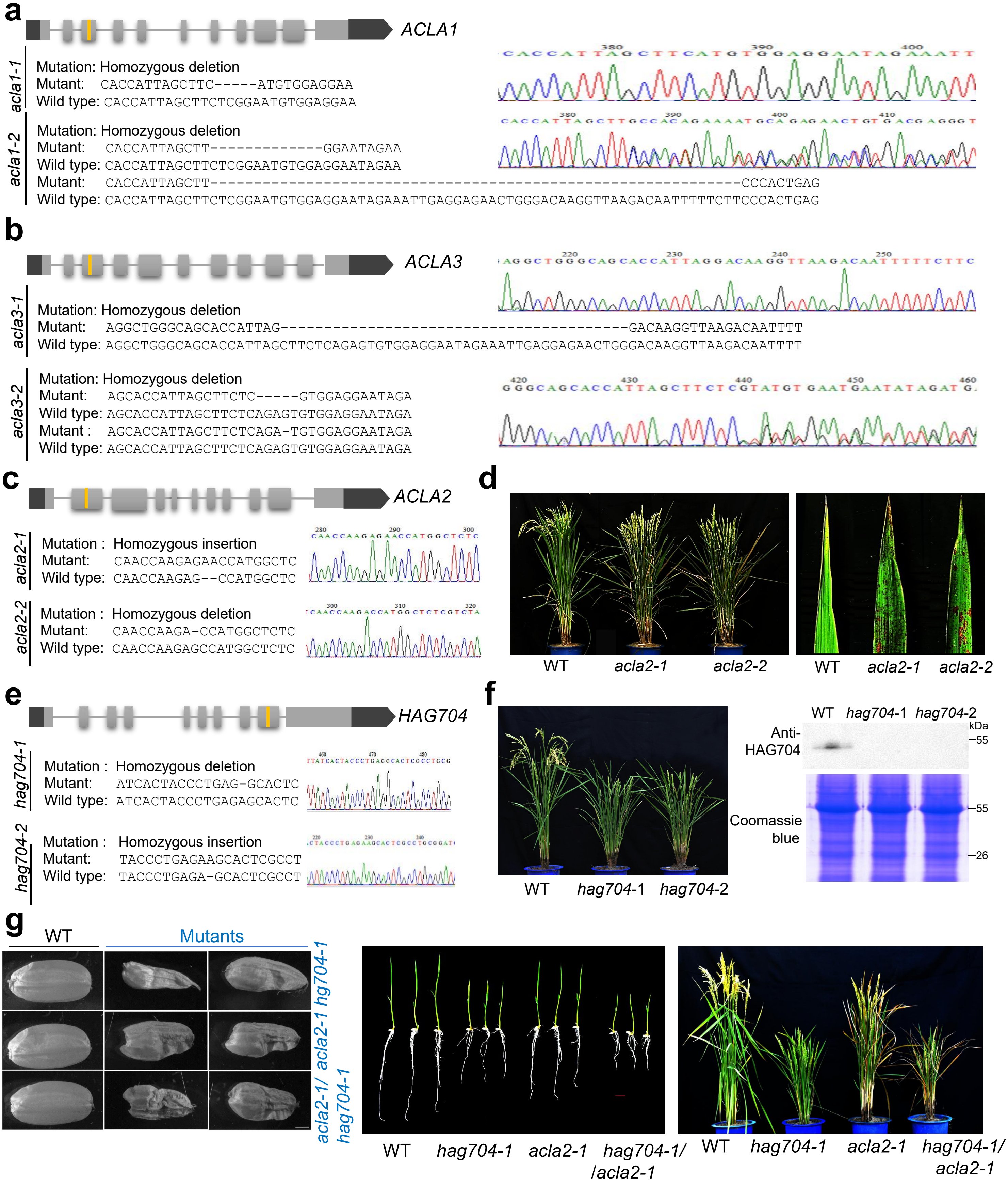

Supplementary Fig. 2. Creation and characterization of *acla1-3* and *hag704* CRISPR/Cas9 mutants. a-c Locus positions of designed ACAL1 (a), ACLA3 (b), and ACLA2 (c) sgRNA and the decoded mutations in two lines for each gene. Vertical yellow lines indicate the mutation positions in the genes. **d** Plant phenotype of *acla2* CRISPR/Cas9 mutants. Left, WT (wild type), *acla2-1*, and *acla2-2* plants at the mature stage. Right, leaf lesion phenotype of *acla2-1* and *acla2-2* mutants. **e** Locus position of designed *HAG704* sgRNA and the decoded mutations in two lines. **f** Plant phenotype of *hag704* CRISPR/Cas9 mutants at the mature stage (left) and immunodetection with anti-HAG704 antibody of HAG704 protein levels in wild type and mutant plants (right). Coomassie blue staining was used as a loading control. The experiments were repeated three times with similar results, and representative data are presented. **g** seed, seedling and mature plant phenotypes of *hag704*/*acla2* double mutant compared with the single mutant and wild type. Source data are provided as a Source Data file.

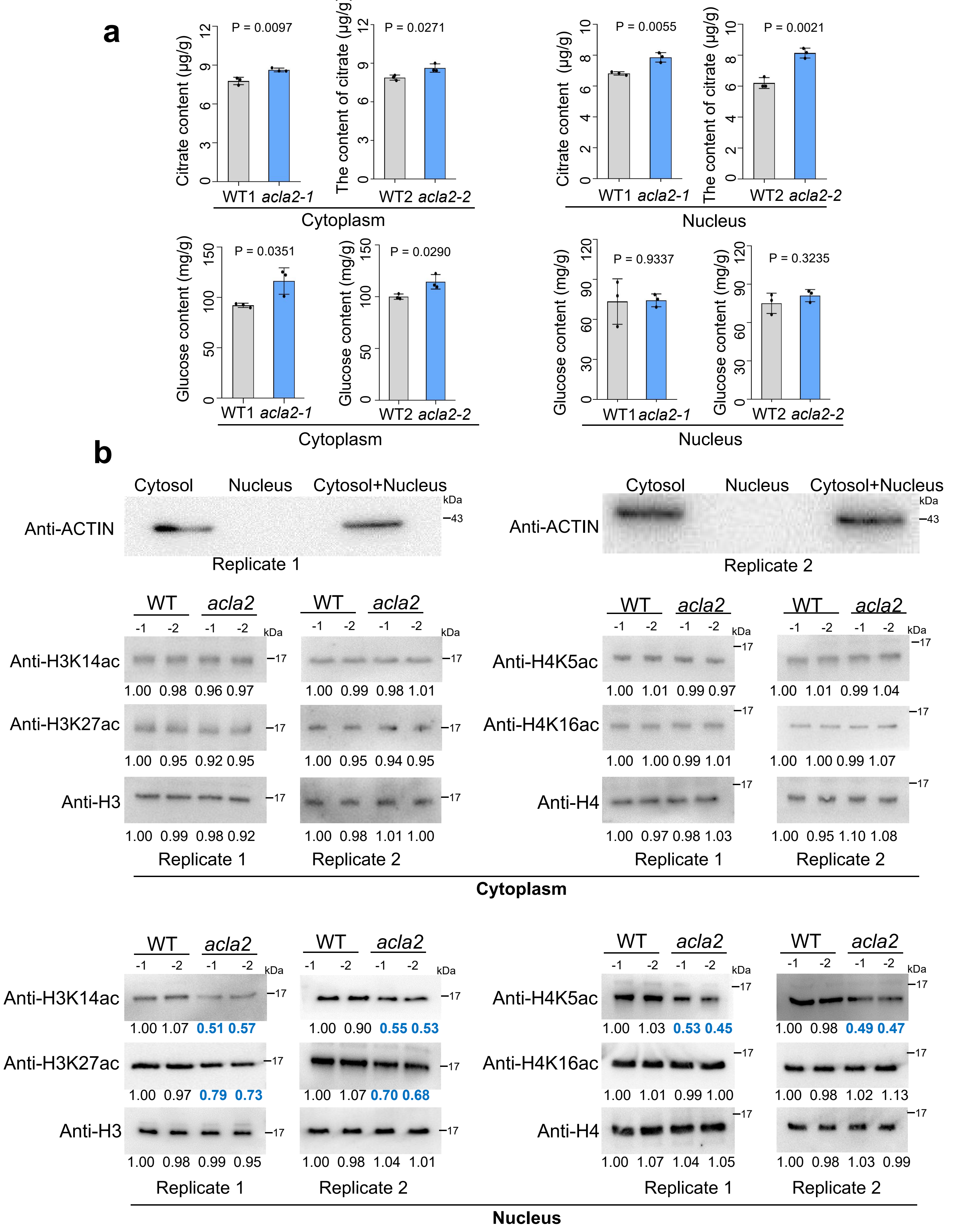

Supplementary Fig. 3. Histone acetylation levels in cytosol and nucleus of *acal2* root cells. a Glucose and citrate levels assay in wild type (WT) and *acla2* (*acla2-1* and *acla2-2*) mutant seedlings (7 d after germination, DAG). Significant difference was calculated by the two-tailed, paired Student *t* test. Error bars represent means ± SD from three biological replicates. **b** Analysis of cytosolic and nuclear histone acetylation levels of *acal2* (*acla2-1* and *acla2-2*) and wild type (WT-1 and WT-2) seedling root cells by immunoblotting. Cytosolic marker ACTIN was used to justify the effectiveness of cell fractions. Two replicates are shown. Immunoblotting bands were quantified using ImageJ and the relative signals indicated below each band were normalized with WT1 set as 1. Clear variations are highlighted in blue. Source data are provided as a Source Data file.

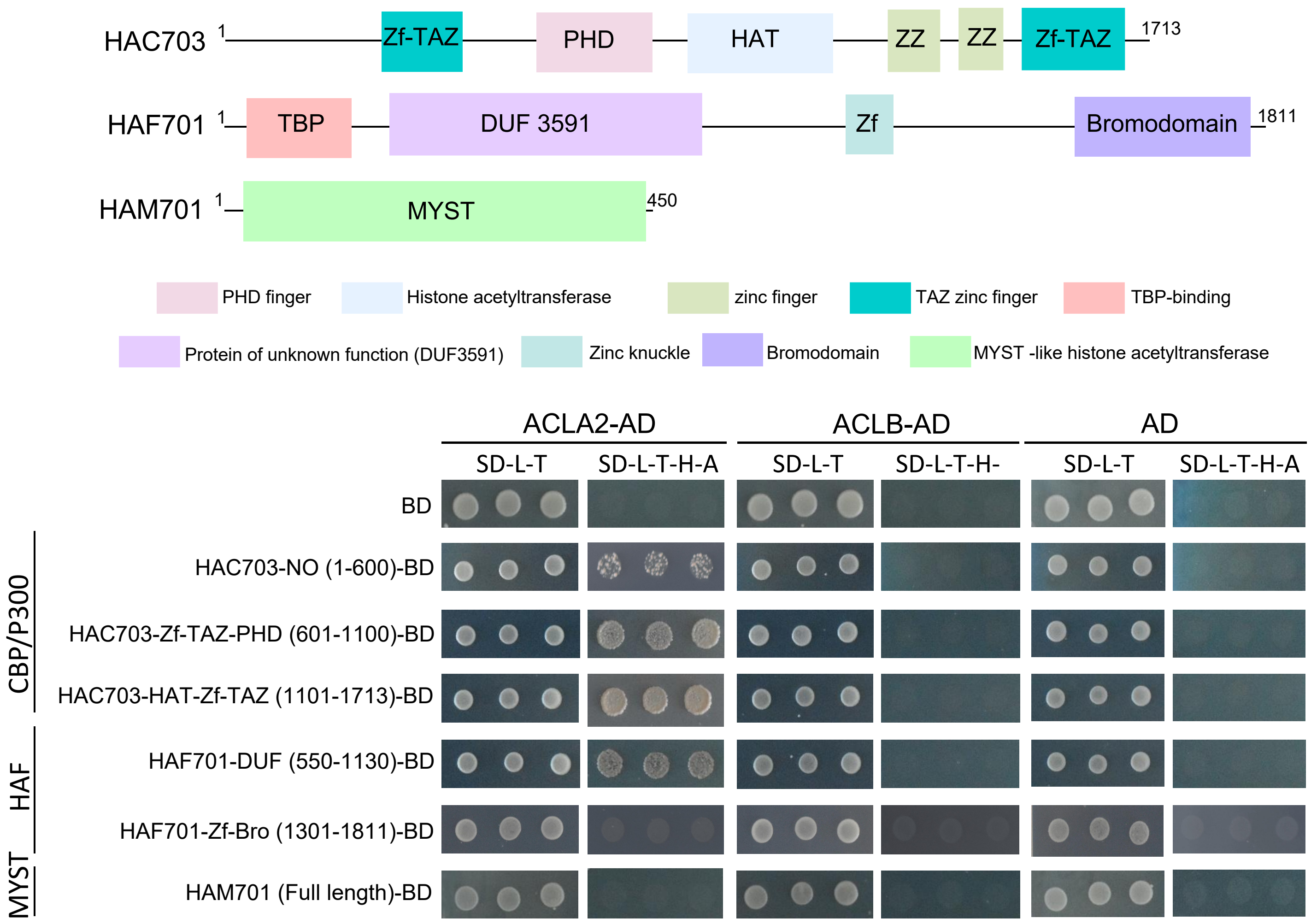

Supplementary Fig. 4. Tests of interaction between ACL and CBP/P300, TAF and MYST HAT members. Upper: Schematics of HAT domains. Lower: Y2H assays of truncated HATs BD (GAL4 binding domain) fusions with ACAL2 and ACLB AD (GAL4 activation domain) fusions in yeast cells. Yeast cells transformed with different combinations of plasmids were grown on the selective SD-LTHA media. Letters indicated the position of amnio acids used for truncation in respective proteins. HAT, histone acetyltransferase. NO, none domain region of HAC703. Zf-TAZ, TAZ zinc finger. PHD, plant homeodomain. Zf, zinc knuckle. Bro, bromodomain. CBP, cAMP-response-element-binding protein. TAF, TATA-binding protein-associated factor. MYST, MOZ, Ybf2/Sas3, Sas2, and Tip60.

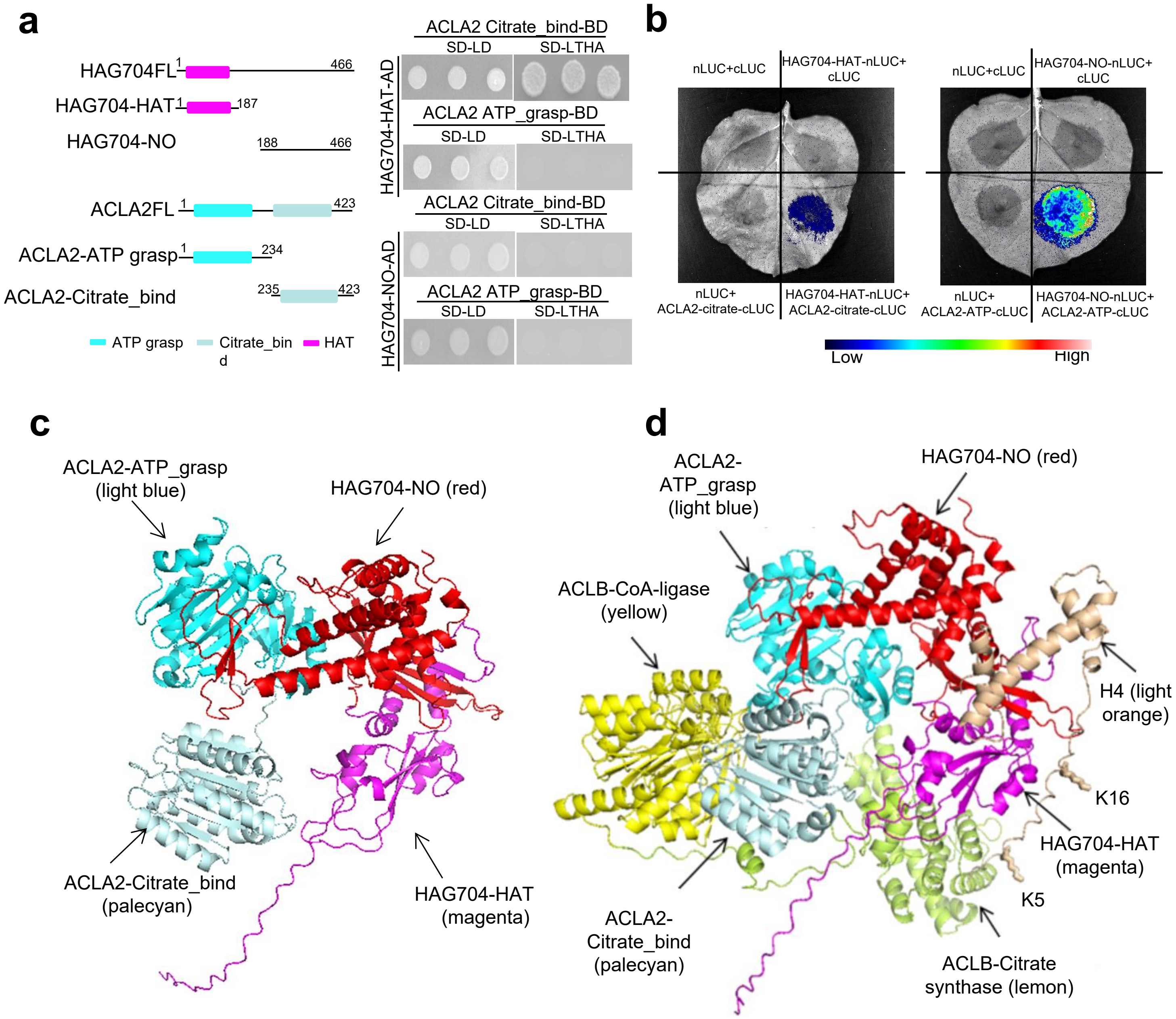

Supplementary Fig. 5. Identification of the interacting regions between HAG704 and ACLA2. a Y2H assays of truncated ACLA2 BD (GAL4 binding domain) fusions with truncated HAG704 AD (GAL4 activation domain) fusions in yeast cells. Schematics of HAG704 and ACLA2 truncated segments are indicated on the left. Yeast cells transformed with different combinations of plasmids were grown on the selective SD-LTHA media. Letters indicated the position of amnio acids used for truncation in respective proteins. **b** BiFC visualization showing interaction between truncated HAG704-nLUC and truncated ACLA2-cLUC proteins in the tobacco epidermal cells. HAG704-HAT (1 aa – 187 aa) is the HAT domain-containing region. HAG704-NO (188 aa – 466 aa) is the none domain region. ACLA2-citrate (235 aa – 423 aa) is the region containing citrate-binding domain. ACLA2-ATP (1 aa – 234 aa) is the region containing ATP grasp domain. **c** Computational modeling of protein interaction between HAG704 and ACLA2. HAG704 non-domain region (NO, red) made direct contacts with the ACLA2 ATP_grasp domain (light blue). HAG704 HAT domain (magenta) interacted with the ACLA2 citrate-binding domain (palecyan). **d** Computer simulation of the complex with histone H4, HAG704, ACLA2 and ACLB proteins. Binding of ACLA2 and ACLB to HAG704 resulted in a steric re-orientation of HAG704, but did not affect the binding of HAG704 to H4. ACLB CoA-ligase domain bound to the citrate-binding domain of ACLA2, while ACLB-citrate synthase domain bound to the HAG704 HAT domain. H4K5 was closer to the citrate synthase domain of ACLB than H4K16 in the spatial structure. Histone H4 is in light orange. The non-domain (NO) of HAG704 is in red, and the HAT domain is in magenta. The ATP_grasp domain of ACLA2 is in light blue, and the Citrate_bind domain is in palecyan. The CoA-ligase domain of ACLB is in yellow, and the Citrate synthase domain is in lemon.

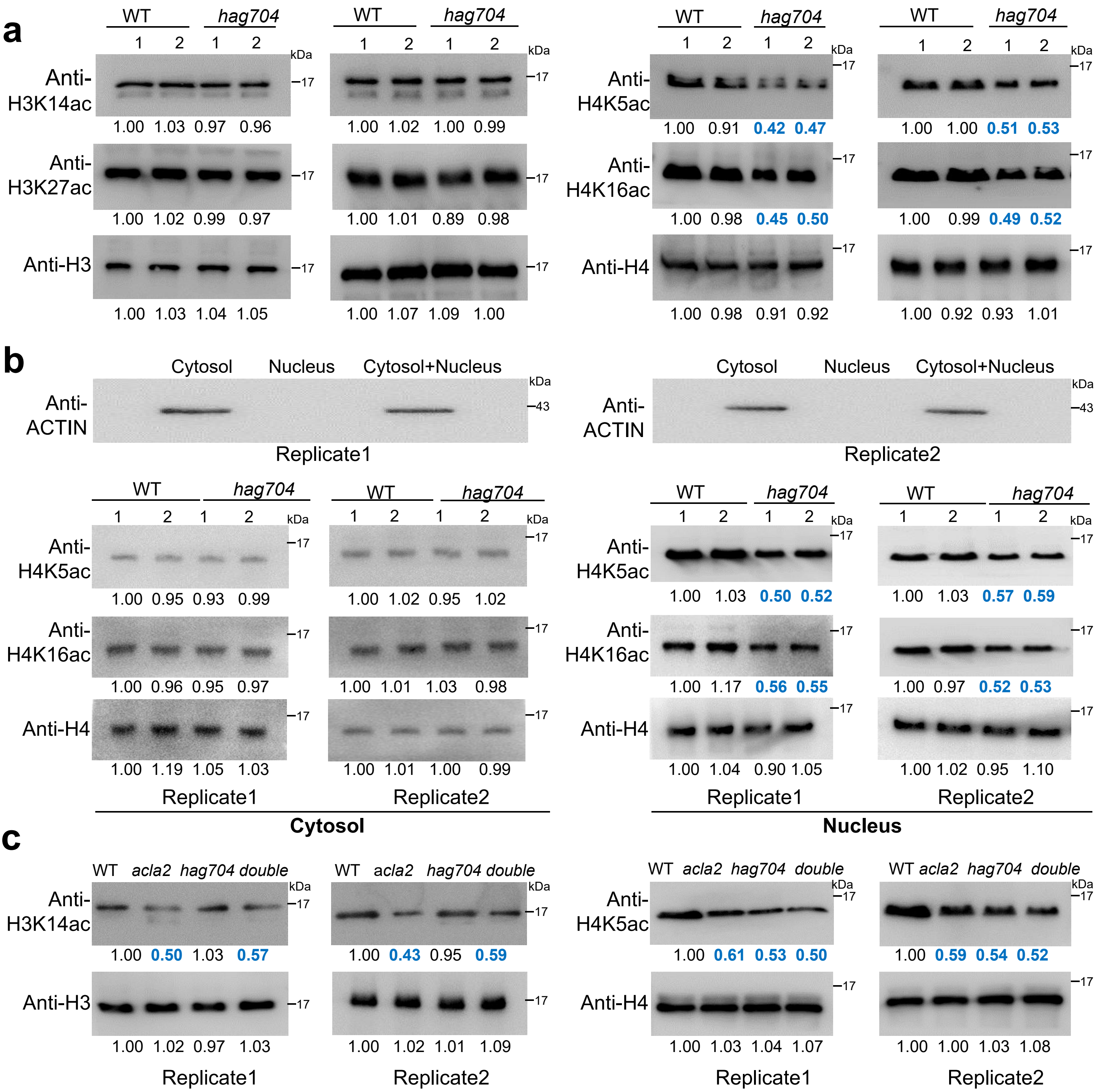

Supplementary Fig. 6. Histone acetylation levels in roots of *hag704* and *hag704*/*acal2* mutants. a Analysis of histone acetylation levels in *hag704* (*hag704-1* and *hag704-2*) and wild type (WT-1 and WT-2) 7 d-old seedling roots by immunoblotting using specific histone H3 and H4 lysine acetylation antibodies as indicated. Total histone extracts were used for the analysis. **b** Analysis of cytosolic and nuclear histone acetylation levels of *hag704* (*hag704-1* and *hag704-2*) and wild type (WT-1 and WT-2) seedling root cells by immunoblotting. Cytosolic marker ACTIN was used to justify the effectiveness of cell fractions. **c** Analysis of histone acetylation levels of *hag704, acl2,* double mutant (*acla2-1/hag704-1*), and wild type plants by immunoblotting. Total histone extracts were used for the analysis. Two replicates are shown. Immunoblotting bands were quantified using ImageJ and the relative signals indicated below each band were normalized with WT1 set as 1. Clear variations are highlighted in blue. Source data are provided as a Source Data file.

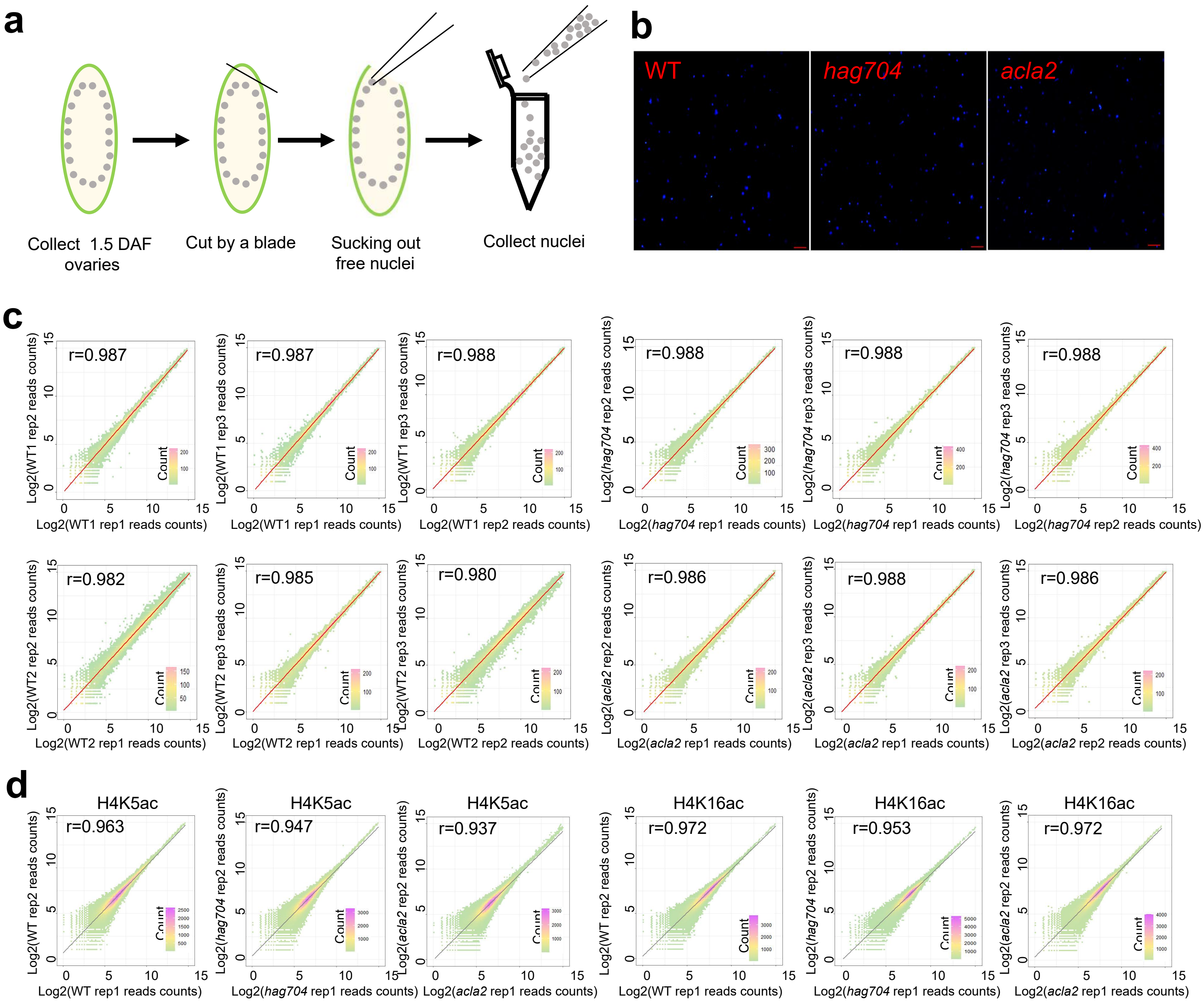

Supplementary Fig. 7. Isolation of syncytial endosperm nuclei for high throughput analysis and multiscatter plots of RNA-seq and CUT&Tag data. a Collection of rice syncytial endosperm nuclei at 1.5 DAF (day after fertilization). Briefly, first cut the ovary at the micropyle end. Then, suck out endosperm nuclei using a glass micropipette. Last, collect the endosperm nuclei in a 1.5 ml RNase-free tubes. **b** DAPI staining of rice 1.5 DAF endosperm nuclei from WT, *hag704*, and *acla2*. Bars = 50 μm. The experiments were repeated three times with similar results, and representative data are presented. **c** Multiscatter plots of endosperm nuclei RNA-seq biological replicates (n=3) for each genotype. Number of mapped reads for each transcript plotted in log scale between two replicates. **d** Multiscatter plots of endosperm nuclei H4K5ac and H4K16ac CUT&Tag (Cleavage Under Targets and Tagmentation) biological replicates (n=2) for each genotype. About ~30, 000 nuclei were used for each replicate for RNA–seq and CUT&Tag. Numbers of the mapped reads from each genomic bin (1 kb) were plotted in log scale between two replicates.

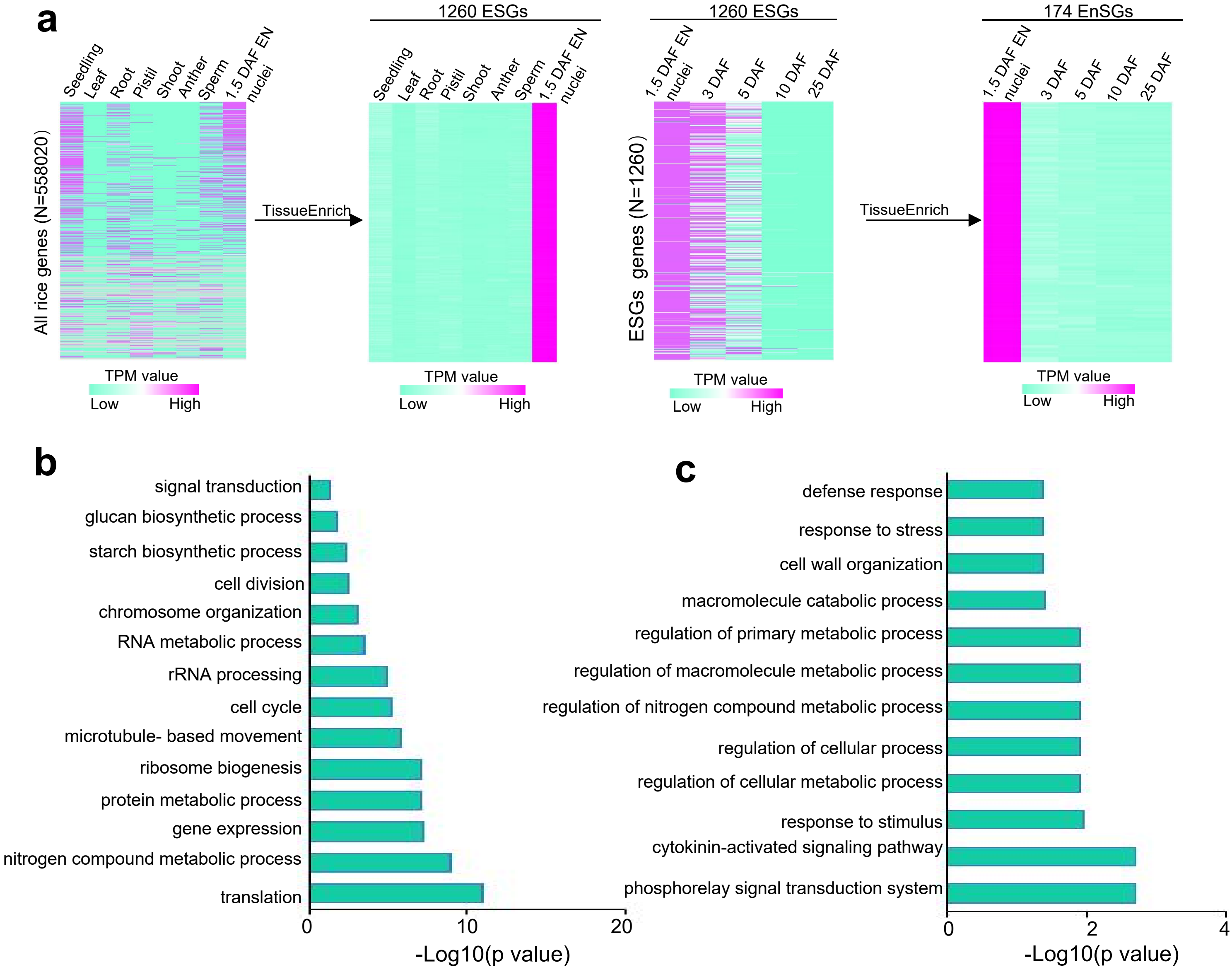

Supplementary Fig. 8. Gene ontology (GO) pathway analysis of 1.5 DAF endosperm nuclei transcriptome. a Identification of endosperm specifically expressed genes (ESGs) and 1.5 DAF syncytial endosperm nuclei specifically expressed genes (EnSGs) in the wild type background. Genes with an expression level greater than or equal to 10 (TPM, transcripts Per Kilobase Million) that also have at least five-fold higher expression levels in a particular tissue compared to the levels in all other tissues are considered tissue enriched. **b** GO pathway analysis of ESGs. **c** GO pathway analysis of EnSGs. For figure 8b and c, p-values are derived from the one-sided Fisher's exact test without multiple comparison correction.

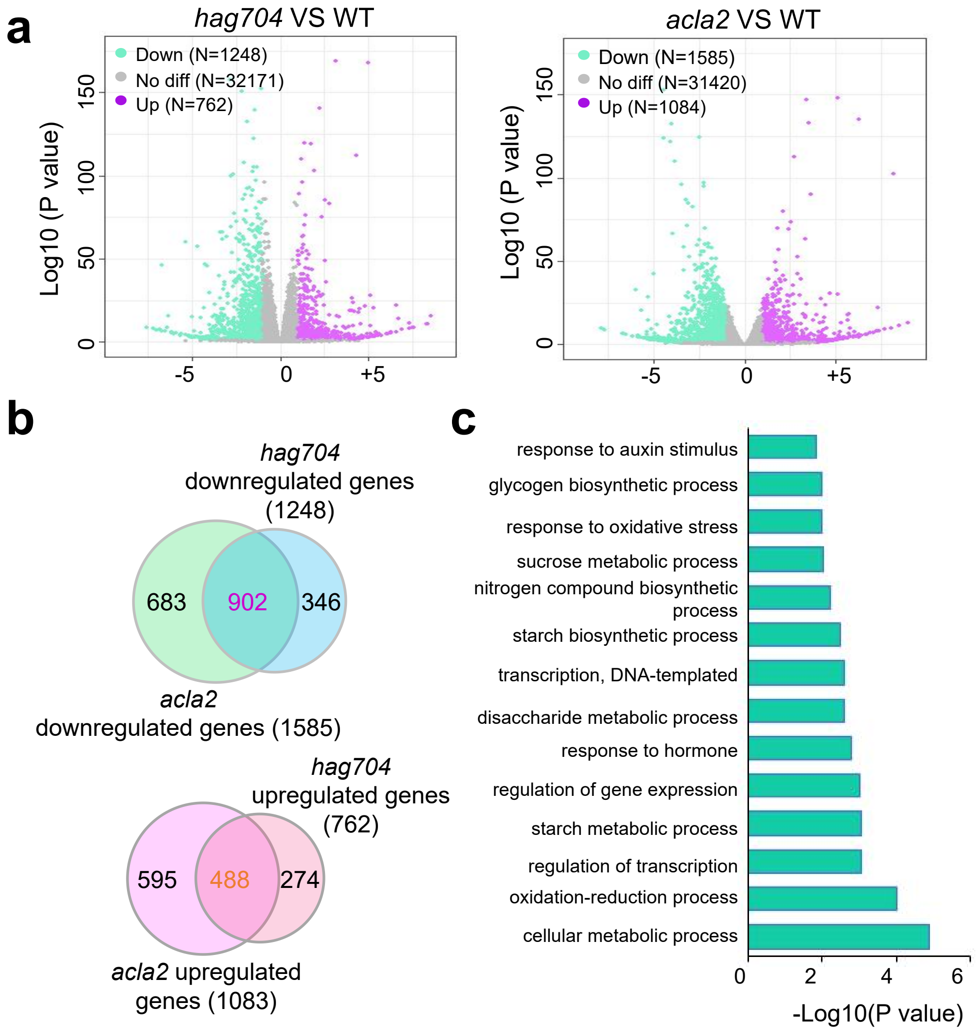

Supplementary Fig. 9. Effects of *hag704* and *acla2* mutations on gene expression in the syncytial endosperm nuclei. a Volcano plots of differential transcript levels in mutants relative to wild type endosperm nuclei. Purple plots represent upregulated genes (fold change > 2, p value < 0.05); cyan plots represent downregulated genes (fold change > 2, p value < 0.05); gray plots represent genes with no significant difference. P-values were derived from a two-sided, unpaired Wald test without multiple comparison correction. **b** Overlap of up- or down-regulated genes in *hag704* and *acla2*. **c** GO pathway analysis of the genes (n=902) that were downregulated in both *acla2* and *hag704* mutants. P-values were derived from the one-sided Fisher's exact test without multiple comparison correction.

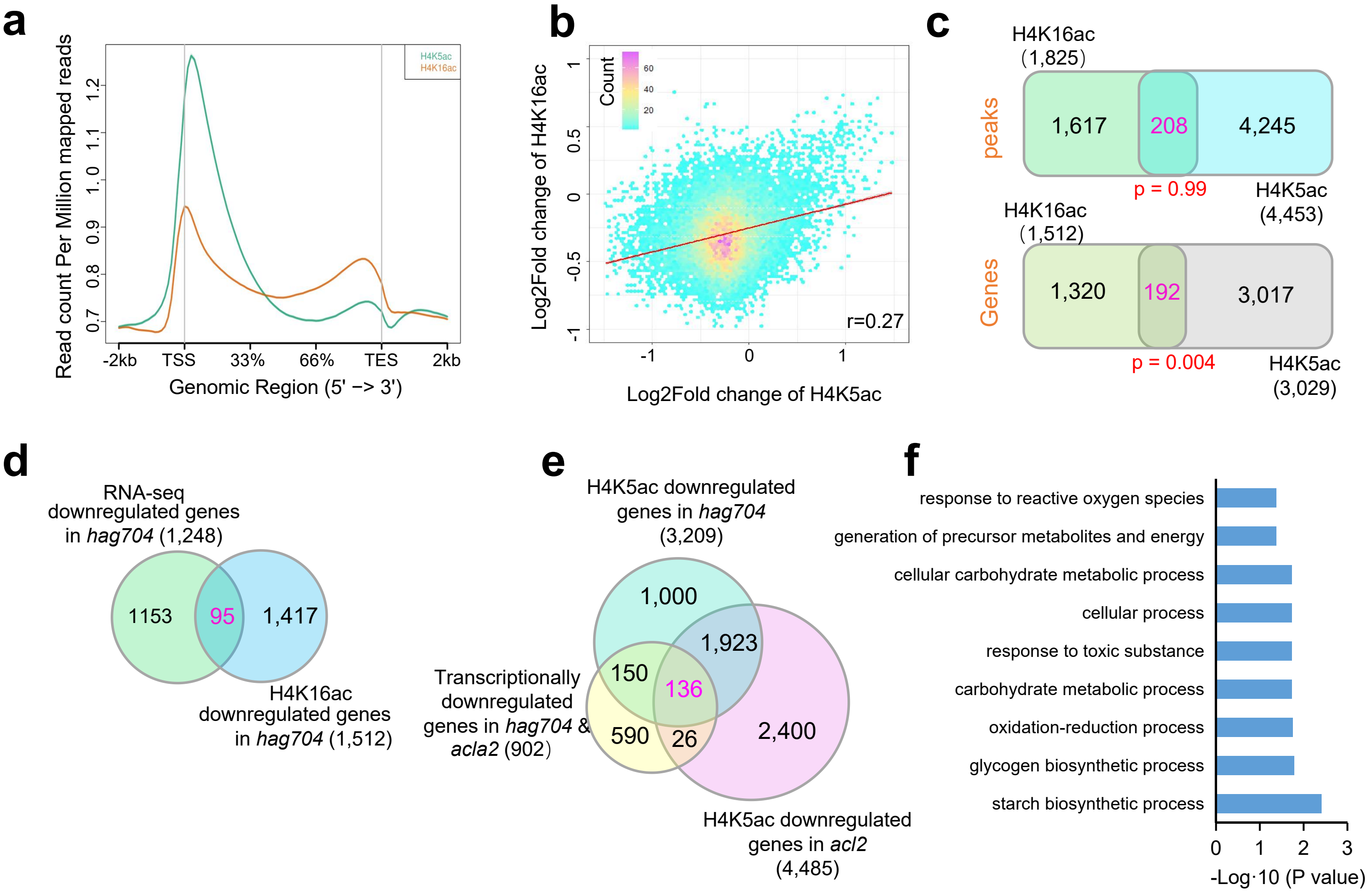

Supplementary Fig. 10. Analysis of endosperm nuclei H4K5 and H4K16 acetylation CUT&Tag data. a Metaplots of wild type 1.5 DAF endosperm nuclei H4K5 and H4K16 acetylation CUT&Tag reads in genes. TSS, transcriptional start site. TES, transcriptional end site. **b** Scattering analysis of H4K5 and H4K16 acetylation changes in *hag704* relative to wild type. Correlation analysis was performed between the differential H4K5ac and H4K16ac peaks (N=18,749) in *hag704* vs WT. Pearson coefficient was shown. **c** Venn diagrams of downregulated H4K5 and H4K16 acetylation peaks (upper) or genes (lower) in *hag704*. P value was calculated by one-sided Fisher's exact test without multiple comparison . **d** Venn diagram between H4K16 hypo-acetylated genes and transcriptionally downregulated genes in *hag704.* **e** Venn diagrams of H4K5 hyp-acetylated genes and transcriptionally downregulated genes in *hag704* & *acla2*. **f** GO pathway enrichment of the 136 genes in (e). P-values were derived from the one-sided Fisher's exact test without multiple comparison correction. Source data are provided as a Source Data file.

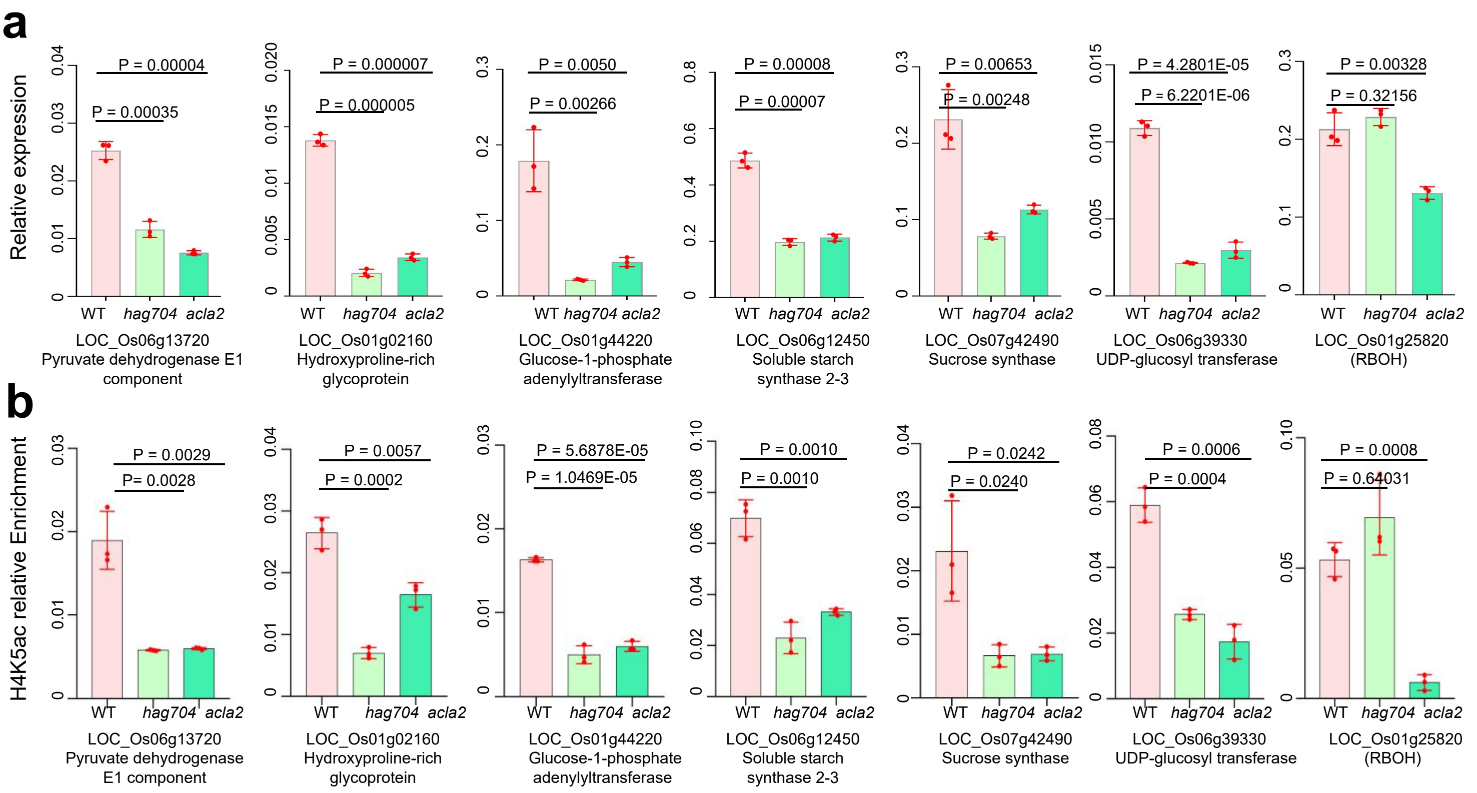

Supplementary Fig. 11. Validation of transcript and H4K5 acetylation levels of downregulated carbon metabolic genes in *acla2* and *hag704* mutants. a qRT-PCR analysis of transcript levels of genes involved in carbon metabolism (relative to *ACTIN* transcripts) in 30-day-old leaves of wild type, *hag704*, and *acla2* plants. **b** ChIP assays with anti-H4K5ac of 30-day-old leaf chromatin isolated from wild type, *hag704*, and *acla2* plants. Bar indicates means ± SD from three replicates. RBOH was selected as a non-carbon gene control. P value was calculated by a two-tailed, paired Student *t* test. Source data are provided as a Source Data file.

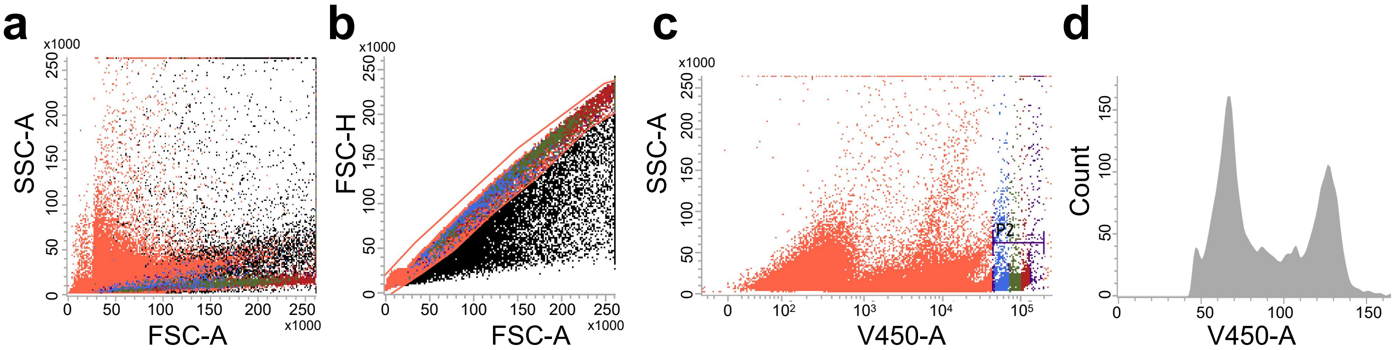

Supplementary Figure 12. Representative gating strategies for analysis of cell cycle of the endosperm nuclei. The gating criteria was as follows: Forward scatter area (FSC-A)/side scatter area (SSC-A) and FSC-A/FSC-H were used to identify the integrity and singlet of nuclei, respectively (a, b). The major endosperm nuclei populations were defined as DAPI-positive (P2, c). Representative ploidy profiles are presented in (d).
