# Supplementary material for: ACL and HAT1 form a nuclear module to acetylate histone H4K5 and promote cell proliferation
Source: Nat Commun. 2023 Jun 5;14:3265. doi: 10.1038/s41467-023-39101-4 (PMC10241871; doi:10.1038/s41467-023-39101-4)
Supplement: Supplementary file 3 — Description of Additional Supplementary File [file 41467_2023_39101_MOESM3_ESM.pdf]

Description of Additional Supplementary Files

Supplementary Data 1: Lists of ESG and EnSG in 1.5 DAF endosperm dividing nuclei.

Supplementary Data 2: Lists of transcriptionally downregulated genes in hag704 and acla2 and their overlaps between two mutants.

Supplementary Data 3: Lists of H4K5 and H4K16 hypo-acetylated genes in hag704 and acla2 and their overlaps.

Supplementary Data 4: Lists of transcriptionally downregulated genes with H4K5 or H4K16 hypo-acetylation in hag704 or acla2.

Supplementary Data 5: Primers used in this study.
