# Supplementary material for: ACL and HAT1 form a nuclear module to acetylate histone H4K5 and promote cell proliferation
Source: Nat Commun. 2023 Jun 5;14:3265. doi: 10.1038/s41467-023-39101-4 (PMC10241871; doi:10.1038/s41467-023-39101-4)
Supplement: Supplementary file 9 — Reporting Summary [file 41467_2023_39101_MOESM9_ESM.pdf]

nature portfolio

Corresponding author(s): Dao-Xiu Zhou

Last updated by author(s): 2023.05.11

Reporting Summary

Nature Portfolio wishes to improve the reproducibility of the work that we publish. This form provides structure for consistency and transparency in reporting. For further information on Nature Portfolio policies, see our Editorial Policies and the Editorial Policy Checklist.

Statistics

For all statistical analyses, confirm that the following items are present in the figure legend, table legend, main text, or Methods section.

n/a	Confirmed	
☐	☒	The exact sample size (*n*) for each experimental group/condition, given as a discrete number and unit of measurement
☐	☒	A statement on whether measurements were taken from distinct samples or whether the same sample was measured repeatedly
☐	☒	The statistical test(s) used AND whether they are one- or two-sided<br>*Only common tests should be described solely by name; describe more complex techniques in the Methods section.*
☒	☐	A description of all covariates tested
☒	☐	A description of any assumptions or corrections, such as tests of normality and adjustment for multiple comparisons
☐	☒	A full description of the statistical parameters including central tendency (e.g. means) or other basic estimates (e.g. regression coefficient) AND variation (e.g. standard deviation) or associated estimates of uncertainty (e.g. confidence intervals)
☐	☒	For null hypothesis testing, the test statistic (e.g. *F*, *t*, *r*) with confidence intervals, effect sizes, degrees of freedom and *P* value noted<br>*Give P values as exact values whenever suitable.*
☒	☐	For Bayesian analysis, information on the choice of priors and Markov chain Monte Carlo settings
☒	☐	For hierarchical and complex designs, identification of the appropriate level for tests and full reporting of outcomes
☐	☒	Estimates of effect sizes (e.g. Cohen's *d*, Pearson's *r*), indicating how they were calculated

Our web collection on statistics for biologists contains articles on many of the points above.

Software and code

Policy information about availability of computer code

Data collection	Blots and luciferase bioluminescence pictures were acquired using the Chemi-Image System (Tanon 5200Multi).<br>Microscopy images were acquired using a light microscope (Nikon Ni-E) equipped with a CCD camera or a laser confocal microscope (Olympus FV1200).<br>All sequencing libraries were prepared in house and raw reads were generated on illumina high-throughtput sequencing platform with manufacture's instruction.
Data analysis	PyMOL (Version 2.5.2), ClustalX (v2.0) and MEGA (v5.2.2) were used for protein structural modeling and phylogenetic tree construction.<br>ImageJ (v1.6.0_24) was used for quantifying immunoblotting results.<br>BD FACSDiva™ software was used for FACS assay.<br>FastP (v0.232), FeatureCounts (version 2.0.3), DESeq2 (v1.36.0), TissueEnrich(1.10.1), Bowtie2 (version 2.3.5.1), samtools (v1.9), MACS software (version 2.2.7.1), deepTools (v2.5.3), IGV (version 2.3.88), DiffBind (v3.5), homer (v4.11), Tbtools (v0.6) and R (v3.5) were used for RNA-seq and CUT&Tag data analysis. Detailed parameters of each of the programs are mentioned in relevant sections in Methods.

For manuscripts utilizing custom algorithms or software that are central to the research but not yet described in published literature, software must be made available to editors and reviewers. We strongly encourage code deposition in a community repository (e.g. GitHub). See the Nature Portfolio guidelines for submitting code & software for further information.

Data

Policy information about availability of data

All manuscripts must include a data availability statement. This statement should provide the following information, where applicable:

- Accession codes, unique identifiers, or web links for publicly available datasets
- A description of any restrictions on data availability
- For clinical datasets or third party data, please ensure that the statement adheres to our policy

The in-house RNAseq data and CUT&Tag data generated in this study have been deposited to the NCBI SRA database under accession code PRJNA874160 [https://www.ncbi.nlm.nih.gov/bioproject/PRJNA874160/]. Other previously published RNAseq data used in this study are available in the NCBI SRA database under accession codes PRJNA13141 [https://www.ncbi.nlm.nih.gov/bioproject/PRJNA13141], PRJNA262032 [https://www.ncbi.nlm.nih.gov/bioproject/PRJNA262032], SRP008821 [https://trace.ncbi.nlm.nih.gov/Traces/?view=study&acc=SRP008821], and in the DDBJ database under the accession codes DRA009458 [https://ddbj.nig.ac.jp/resource/sra-submission/DRA009458] and DRA007969 [https://ddbj.nig.ac.jp/resource/sra-submission/DRA007969]. The structure of human ACL was downloaded from the PDB database under the accession number:6HXH [https://www.rcsb.org/structure/6HXH]. Extra data for the individual measurements are available on request. The source data for Figs. 1a, d-g, 2a, 5d, 6b, c, 7c, and Supplementary Figs. 3a, 10b, 11 are provided in a Source Data file. This file also includes uncropped and unprocessed scans of the western blots for Figs. 2b, c, 3d, f, g, 4, and Supplementary Figs. 2f, 3b, 6. Source data are provided with this paper.

Human research participants

Policy information about studies involving human research participants and Sex and Gender in Research.

Reporting on sex and gender	N/A
Population characteristics	N/A
Recruitment	N/A
Ethics oversight	N/A

Note that full information on the approval of the study protocol must also be provided in the manuscript.

Field-specific reporting

Please select the one below that is the best fit for your research. If you are not sure, read the appropriate sections before making your selection.

☒ Life sciences ☐ Behavioural & social sciences ☐ Ecological, evolutionary & environmental sciences

For a reference copy of the document with all sections, see nature.com/documents/nr-reporting-summary-flat.pdf

Life sciences study design

All studies must disclose on these points even when the disclosure is negative.

Sample size	Three biological replicates were performed for RNA-seq and two biological replicates were performed for CUT&Tag-seq in this study. For phenotype checking and enzyme activity assay, at lest two biological replicates were performed in this study.
Data exclusions	No data were excluded from analysis.
Replication	At lest two biological replicates were performed for experiments in this study as indicated in the figure legends.
Randomization	Samples were randomly allocated into experimental groups according to different mutants.
Blinding	Blinding was not relevant to our study.

Reporting for specific materials, systems and methods

We require information from authors about some types of materials, experimental systems and methods used in many studies. Here, indicate whether each material, system or method listed is relevant to your study. If you are not sure if a list item applies to your research, read the appropriate section before selecting a response.

Materials & experimental systems

n/a	Involved in the study
☐	☒ Antibodies
☒	☐ Eukaryotic cell lines
☒	☐ Palaeontology and archaeology
☒	☐ Animals and other organisms
☒	☐ Clinical data
☒	☐ Dual use research of concern

Methods

n/a	Involved in the study
☐	☒ ChIP-seq
☐	☒ Flow cytometry
☒	☐ MRI-based neuroimaging

Antibodies

Antibodies used

Antibodies used in this study:
anti-H3K9ac: Rabbit polyclonal antibody, Millipore (07-352). Dilution: 1:1000.
anti-H3K14ac: Rabbit monoclonal antibody, Abcam (ab52946). Dilution: 1:1000.
anti-H3K18ac: Rabbit polyclonal antibody, Millipore (07-354). Dilution: 1:1000.
anti-H3K27ac: Rabbit polyclonal antibody, Abcam (ab4729). Dilution: 1:1000.
anti-H3: Rabbit polyclonal antibody, Abcam (ab1791). Dilution: 1:1000.
anti-H3Kac: Rabbit polyclonal antibody, Millipore (17-615). Dilution: 1:1000.
anti-H4: Rabbit monoclonal, Abcam (ab177840). Dilution: 1:1000.
anti-H4K5ac: Rabbit polyclonal antibody, Millipore (07-327). Dilution: 1:1000.
anti-H4K12ac: Rabbit polyclonal antibody, PTM-Biolab (PTM-121). Dilution: 1:1000.
anti-H4K16ac: Rabbit polyclonal antibody, Millipore (07-329). Dilution: 1:1000.
anti-H4Kac: Rabbit polyclonal antibody, Millipore (06-598). Dilution: 1:1000.
anti-GFP: Rabbit polyclonal antibody, Abcam (ab290). Dilution: 1:1000.
Anti-6X His: Rabbit monoclonal antibody, Abcam (ab213204). Dilution: 1:1000.
anti-GST: Rabbit monoclonal antibody, Abcam (ab19256). Dilution: 1:1000.
anti-FLAG: Mouse monoclonal antibody, Sigma (F3165). Dilution: 1:1000.
anti-Rabbit IgG: Goat anti-rabbit IgG (H+L) cross-adsorbed secondary Antibody, Alexa Fluor™ 568, Invitrogen (A-11011), Dilution: 1:200.
anti-Rabbit IgG: HRP goat anti-Rabbit IgG, Abbkine (A21020). Dilution: 1:10000.
anti-Mouse IgG: HRP goat anti-Mouse IgG, Abbkine (A21010). Dilution: 1:10000.
anti-ACLA2: Homemade, no catalog number available. Dilution: 1:1000 (Western blot) or 1:200 (Immunocytochemistry).
anti-HAG704: Homemade. no catalog number available. Dilution: 1:1000 (Western blot) or 1:200 (Immunocytochemistry).

Validation

anti-H3K9ac (Millipore, 07-352): https://www.merckmillipore.com/CN/zh/product/Anti-acetyl-Histone-H3-Lys9-Antibody,MM_NF-07-352
anti-H3K14ac (Abcam, ab52946): https://www.abcam.com/Histone-H3-acetyl-K14-antibody-EP964Y-ChIP-Grade-ab52946.html
anti-H3K18ac (Millipore, 07-354): https://www.merckmillipore.com/CN/zh/product/Anti-acetyl-Histone-H3-Lys18-Antibody,MM_NF-07-354
anti-H3K27ac (Abcam, ab4729): https://www.abcam.com/histone-h3-acetyl-k27-antibody-chip-grade-ab4729.html
anti-H3 (Abcam, ab1791): https://www.abcam.com/histone-h3-antibody-nuclear-marker-and-chip-grade-ab1791.html
anti-H3Kac (Millipore, 17-615): https://www.merckmillipore.com/CN/zh/product/ChIPAb-Acetyl-Histone-H3-ChIP-Validated-Antibody-and-Primer-Set,MM_NF-17-615
anti-H4 (Abcam, ab177840): https://www.abcam.cn/histone-h4-antibody-epr16599-chip-grade-ab177840.html
anti-H4K5ac (Millipore, 07-327): https://www.merckmillipore.com/CN/zh/product/Anti-acetyl-Histone-H4-Lys5-Antibody,MM_NF-07-327
anti-H4K12ac (PTM-Biolab, PTM-121): https://www.ptmbiolabs.com/product/ptm-121/
anti-H4K16ac (Millipore, 07-329): https://www.merckmillipore.com/CN/zh/product/Anti-acetyl-Histone-H4-Lys16-Antibody,MM_NF-07-329
anti-H4Kac (Millipore, 06-598): https://www.merckmillipore.com/CN/zh/product/Anti-acetyl-Histone-H4-Antibody,MM_NF-06-598
anti-FLAG (Sigma, F3165): https://www.sigmaaldrich.com/JP/ja/product/sigma/f3165
anti-GFP (Abcam, ab290): https://www.abcam.com/GFP-antibody-ab290.html
anti-6X His tag (Abcam, ab213204): https://www.abcam.com/6x-his-tag-antibody-epr20547-chip-grade-ab213204.html
anti-GST antibody (Abcam, ab19256): https://www.abcam.com/gst-antibody-ab19256.html
Anti-ACLA2 and anti-HAG704 antibodies were validated by manufacturer and data provided in this paper.
anti-Rabbit IgG: https://www.thermofisher.com/antibody/product/Goat-anti-Rabbit-IgG-H-L-Cross-Adsorbed-Secondary-Antibody-Polyclonal/A-11011
anti-Rabbit IgG: https://www.abbkine.com/product/hrp-goat-anti-rabbit-igg-a21020/
anti-Mouse IgG: https://www.abbkine.com/product/hrp-goat-anti-mouse-igg-a21010/

ChIP-seq

Data deposition

☒ Confirm that both raw and final processed data have been deposited in a public database such as GEO.

☒ Confirm that you have deposited or provided access to graph files (e.g. BED files) for the called peaks.

Data access links<br>*May remain private before publication.*	All of raw data has been deposited to the NCBI SRA database under accession code PRJNA874160 [https://www.ncbi.nlm.nih.gov/bioproject/PRJNA874160/].
Files in database submission	WT_Rep1_k16<br>WT_rep2_k16<br>ACL_rep1_k16<br>ACL_rep2_K16<br>HAG_rep1_K16<br>HAG_rep2_k16<br>IgG<br>K5_ACL_rep2<br>K5_ACL2_rep1<br>K5_HAG_rep2<br>K5_HAG704_rep1<br>K5_WT_rep1<br>K5_WT_rep2
Genome browser session<br>(e.g. UCSC)	Not available.

Methodology

Replicates	Two biological replicates for each histone mark (H4K5ac and H4K15ac) in examined tissues.
Sequencing depth	About 20 million pair-end (2x150bp) raw reads on average for each experiment.
Antibodies	anti-H4K5ac (Millipore, 07-327) and anti-H4K16ac (Millipore, 07-329).
Peak calling parameters	macs2 callpeak -f BAMPE -B -q 0.05 -g 3.6e+8
Data quality	Alll identified peaks in the study were called with a qval threshold of 0.05(FDR 5%).
Software	FastP (v0.232), Bowtie2 (version 2.3.5.1), samtools (v1.9), MACS software (version 2.2.7.1), deepTools (v2.5.3), IGV (version 2.3.88), DiffBind (v3.5), homer (v4.11).

Flow Cytometry

Plots

Confirm that:

☒ The axis labels state the marker and fluorochrome used (e.g. CD4-FITC).

☒ The axis scales are clearly visible. Include numbers along axes only for bottom left plot of group (a 'group' is an analysis of identical markers).

☒ All plots are contour plots with outliers or pseudocolor plots.

☒ A numerical value for number of cells or percentage (with statistics) is provided.

Methodology

Sample preparation	Syncytial endosperm nuclei were isolated at 1.5 DAF, labeled with DAPI (4′, 6-diamidino-2-phenylindole dihydrochloride) in PBS (pH 7.5).
Instrument	BD FACSCelest, USA
Software	BD FACSDiva™ software
Cell population abundance	Cell population abundance is determined by BD FACSDiva™ software (USA). For cell cycle analysis, we acquired at least 10,000 events.

Gating strategy

The FACS assay used in this study is an established protocol for cell cycle analysis. DAPI stained leaf nuclei sample were used as a 2N reference

☒ Tick this box to confirm that a figure exemplifying the gating strategy is provided in the Supplementary Information.
